# Psychometric Properties of the Chinese (Putonghua) Version of the Oxford Cognitive Screen (OCS-P) in Subacute Poststroke Patients without Neglect

**DOI:** 10.1155/2018/6827854

**Published:** 2018-05-21

**Authors:** Wen-jun Hong, Jing Tao, Alex W. K. Wong, Shan-li Yang, Man-tak Leung, Tatia M. C. Lee, Nele Demeyere, Stephen C. L. Lau, Chi-wen Chien, Chetwyn C. H. Chan, Li-dian Chen

**Affiliations:** ^1^College of Rehabilitation Medicine, Fujian University of Traditional Chinese Medicine, Fuzhou, China; ^2^Fujian Key Laboratory of Rehabilitation Technology, Fuzhou, China; ^3^Fujian Provincial Rehabilitation Industrial Institution, Fuzhou, China; ^4^Program in Occupational Therapy, Washington University School of Medicine, St. Louis, USA; ^5^Department of Neurology, Washington University School of Medicine, St. Louis, USA; ^6^Affiliated Rehabilitation Hospital, Fujian University of Traditional Chinese Medicine, Fuzhou, China; ^7^Department of Chinese Bilingual Studies, The Hong Kong Polytechnic University, Hong Kong; ^8^Neuropsychology Laboratory, Department of Psychology, The University of Hong Kong, Hong Kong; ^9^Cognitive Neuropsychology Centre, Department of Experimental Psychology, University of Oxford, UK; ^10^Applied Cognitive Neuroscience Laboratory, Department of Rehabilitation Sciences, The Hong Kong Polytechnic University, Hong Kong; ^11^Department of Rehabilitation Sciences, The Hong Kong Polytechnic University, Hong Kong; ^12^Fujian University of Traditional Chinese Medicine, Fuzhou, China

## Abstract

**Background:**

Oxford Cognitive Screen is designed for assessing cognitive functions of poststroke patients. This study was aimed to assess the psychometric properties of the Chinese (Putonghua) version of the Oxford Cognitive Screen-Putonghua (OCS-P) for use among poststroke patients without neglect.

**Methods:**

Expert review panel evaluated content validity of the Chinese-translated items. After pilot tested the translated items, the patients and healthy participants completed the OCS-P as well as the Montreal Cognitive Assessment (MoCA-ChiB) and Goldenberg's test. A group of patients completed OCS-P for the second time within seven days. Data analyses included confirmatory factor analysis, item difficulty and item-total correlation, inter- and intrarater reliability, internal consistency, and between-group discrimination.

**Results:**

One hundred patients and 120 younger (*n* = 60) or older (*n* = 60) healthy participants completed all the tests. Modifications were required for items in the “Picture Naming”, “Orientation”, and “Sentence Reading” subscales. Confirmatory factor analysis revealed a three-factor structure for the OCS-P subscales. The internal consistency coefficients for the three identified test dimensions were 0.30 to 0.52 (Cronbach's alpha). Construct validity coefficients between the OCS-P and MoCA-ChiB subscales were 0.45 < *r* < 0.79 (*p* < 0.001) and the “Praxis” subscale of OCS-P and Goldenberg's test was *r* = 0.72 (*p* < 0.001). The interrater reliability coefficients for the subscales were in general higher than the intrarater reliability coefficients. The “Picture Naming” and “Numerical Cognition” subscales were the most significant (*p* = 0.003) for differentiating patient participants from their older healthy counterpart.

**Conclusion:**

This study generated satisfactory evidence on the content validity, substantive validity, construct validity, inter- and intrarater reliability, and known-group discrimination of the OCS-P. They support its application among poststroke patients who speak Putonghua. Future studies could review the existing five-dimension domains for improving its structural validity and internal consistency as well as generate evidence of the OCS-P for use among the poststroke patients with neglect.

## 1. Introduction

Cerebrovascular disease or stroke represents a major cause of morbidity and mortality in older individuals [1]. Substantial evidence indicates that poststroke survivors commonly suffer from different types of cognitive impairments [2–5]. Aphasia, visual disorders, attention, and executive dysfunction are common problems among poststroke survivors [6]. The severity of cognitive impairments has been found to be a major predictor of rehabilitation outcomes [7–11]. A valid measurement specific for the identification of cognitive deficits in poststroke survivors is critical for designing and evaluating effective stroke rehabilitation treatments. Existing instruments for screening cognitive functions in poststroke survivors include the Mini Mental State Examination (MMSE), Montreal Cognitive Assessment (MoCA), and Cambridge Cognitive Examination (CAMCOG) [12, 13]. However, these instruments were not targeted for assessments of post-stroke-specific cognitive impairments. Indeed, several studies have reported the flaws of these instruments for use in assessments of such impairments. The MMSE was not sufficiently sensitive for identifying impaired abstract reasoning, executive functioning, or visual perception/construction [14, 15]. The MoCA has been found to suffer from low sensitivity in screening for poststroke cognitive deficits [16, 17]. To date, no strong evidence has been reported to support the utility of CAMCOG for assessing cognitive deficits in poststroke survivors [18–20].

The Oxford Cognitive Screen (OCS) is designed to serve as a rapid screening tool for identification of post-stroke-specific cognitive impairments [6]. The OCS consists of five domains: language, Numerical Cognition and Praxis, memory, attention, and executive function; and these domains are further subcategorized into ten subscales [6]. OCS is superior to other cognitive screening tools for poststroke patients, such as the MMSE [21] and MoCA [22], because it provides a domain-specific assessment, including measures for common stroke-specific cognitive problems such as apraxia, dysphasia, and neglect. The administration of OCS takes approximately 15 minutes.

The original OCS was found to have satisfactory concurrent validity with other cognitive measures and test-retest reliability [6]. The spatial attention and executive function subtests of the OCS were reported to predict the long-term functional capabilities of poststroke patients [23]. Psychometric properties of the Hong Kong version of OCS (HK-OCS) included sound concurrent validity, excellent intrarater and interrater reliability, fair test-retest reliability, and acceptable internal consistency (all 10 subtests). Semantic, episodic memory, number writing, and orientation were significant predictors of functional outcomes of Cantonese speaking chronic stroke survivors [24]. Study on the Italian version of OCS explored the influence of demographic characteristics on the test results [25]. Age, education, and to a lesser extent gender of poststroke patients were revealed affecting the scores of selected subscales. The authors proposed setting age-, education-, and gender-adjusted norms for use of OCS in Italy.

Validation study on the Chinese (Putonghua) version of the OCS (called OCS-P) is necessary for three reasons. First, differences in linguistics and semantics between the English and Chinese languages could substantially reduce the content validity of OCS-P. Second, differences in culture and everyday lifestyle could impact the interpretability and difficulty level of the text; for example, there may be differences related to the naming of pictures. Third, the OCS is relatively new in the field for which different validation studies are needed to demonstrate its robust psychometric properties for use among poststroke patients. As the number of poststroke patients with neglect is substantially fewer than those without neglect in the subacute rehabilitation, this study primarily focused on patients without neglect to adequately power the demonstration of validation evidence. The aim of this study therefore was to test the psychometric properties of the OCS-P for use among poststroke patients without neglect. Types of evidence include content validity, structural validity, substantive validity, construct validity, internal consistency, inter- and intrarater reliability, and known-group differences. This paper adopted a hypothesis testing approach for guiding data analysis and interpretation of the results [26]. We hypothesized that the OCS-P would have good interrater reliability and, to a lesser extent, intrarater reliability based on the standardized test administration procedures. For validity, it was hypothesized that the test structure of OCS-P would be multidimensional of which is similar to the five-domain proposed in the original OCS. Selected subscales of the OCS-P would form moderate to strong relationships with the criterion instruments, which reflect good construct validity. Poststroke patients would obtain lower scores than their healthy older counterpart on selected OCS-P subscales.

## 2. Methods

### 2.1. Participants

The three groups of participants were one group of poststroke patients, one group of older healthy adults, and one group of younger adults. Inclusion criteria for the poststroke patients recruited in this study were the following: (1) brain lesions caused by stroke that were confirmed by CT or MRI; (2) first stroke occurred in the previous 3 months; (3) the patient exhibited cognitive impairments resulting from the stroke; (4) the patient exhibited no detectable symptoms of visual neglect as screened with Albert's test [27]; and (5) informed consent to participate in the study was provided. Exclusion criteria were as follows: (1) history of depressive mood or other mental disorders as screened with the Beck Depression Inventory-II [28, 29] and (2) inability to follow verbal instructions. The screening tests used were to reduce the heterogeneity of the patient group. Mood would influence participants' performance on the OCS in general while visual neglect would influence performances on OCS-P subscales involving visual perception such as in the “Visual Field Test” and “Broken Heart Test” subscales. The cut-off age between younger and older healthy adult participants was set at 40 years. The reason for setting this cut-off was that a trend of younger age adults (in 40's and 50's) were found to suffer from stroke [30] and the utility of the OCS therefore will need to cater patients within a wider age range. For the older healthy participants, they were relatives or caregivers of the participating patients with comparable age and level of education. Other inclusion criteria for the healthy participants were as follows: (1) no noticeable physical or mental disabilities; (2) MoCA score > 25; (3) no known history of neurological or psychiatric diseases; (4) no known history of alcoholism or substance abuse; and (5) provided informed consent to participate in the study. All participants spoke Putonghua, the official language of China, as their mother language. Ethics approval for this study was granted by the institutional review board at the study institution.

### 2.2. Content Validation

The task descriptions, instructions in the test manual, and scoring criteria of the OCS were translated into Chinese (Putonghua) by qualified bilingual translators who were not familiar with the instrument. All items followed the same translation process, except for the “Sentence Reading” and “Delayed Recall and Recognition” subscales. Because these subscales involved Chinese linguistics, a linguist and speech pathologist were invited to construct the Putonghua version. An expert panel was formed to evaluate the content equivalence (semantic meaning), fluency, relevance, and representativeness of Chinese-translated items. The panel review format and protocol were in compliance with our previous studies [31–33]. The expert panel was composed of five bilingual (English and Chinese) physical medicine specialists who had at least 15 years of experience in neurorehabilitation. Researchers explained the purpose of OCS and described the review procedures to the panel. All panel members had access to the original English and translated Putonghua versions. Panel members were guided by a structured guide, including closed-ended questions followed by open-ended questions to review the equivalence and fluency of each item and then the relevance and representativeness of the content. After that, during group discussion each member was asked to raise concerns about the translated version and suggest possible changes. Any change to the translated version was confirmed by consensus among all members. The entire session lasted for six hours. A pilot field test of the translated version was conducted for collecting patients' feedback on the level of fluency and understandability of the item content before the main study. The scores on the subscales and the feedback collected from the patients would guide revision of the items for producing the final set of items for the OCS-P.

### 2.3. Procedure

For the pilot field test, the translated OCS items were administered to poststroke patients recruited via convenience sampling. After completing the OCS-P, the patients were asked to provide feedback on the level of fluency and understandability of the instrument. Their feedback was recorded verbatim by the lead investigator. For the main study, patients and healthy participants were screened according to the inclusion and exclusion criteria by a single researcher (the first author) who was a specialist in physical medicine. Demographics of participants were abstracted from medical records. Each patient completed the OCS-P followed by the Goldenberg's test and Chinese (Beijing) version MoCA (MoCA-ChiB) within seven days after the screening by the first author. The same sequence of test administration was used for all patients. To avoid fatigue, a 30-minute break was given between the administration of OCS-P and the two subsequent criterion tests. Healthy participants only completed the OCS-P. Fifteen of the patient participants were randomly selected for the test-rest and interrater reliability testing. To establish interrater reliability, the second rater had undergone training to administer the OCS-P by the lead investigator. A second rater observed the test administration and scored the patient's performance without communicating with the first rater. To establish intrarater reliability, the OCS-P was administered to the same 15 patients seven days after the first test administration.

### 2.4. Instruments

#### 2.4.1. OCS

The OCS [6] is a rapid screening tool for identifying post-stroke-specific cognitive impairments. There are ten subscales covering five different cognitive domains (attention and executive function, language, memory, number processing, and Praxis). The subscales are “Picture Naming”, “Semantics”, “Orientation”, “Visual Field”, “Sentence Reading”, “Number-Number Writing” and “Number-Calculation”, “Broken Hearts Test”, “Praxis” (or called Imitation), “Delayed Recall and Recognition”, and “Executive Task”. Performances on items in each subscale yield a subscale score according to the scoring standards described in the test manual. OCS was found to have fair to good convergent validity (*r* = −0.35 to 0.72) and test-retest reliability (ICC = 0.331 to 0.776) and good sensitivity (from 27.6% to 94.1%) and specificity (ranged from 70.1% to 98.3%) [6].

#### 2.4.2. MoCA-ChiB

The original version of the MoCA was developed as a screening tool for cognitive impairment [22]. The test items are grouped under eight domains of cognitive functioning. The MoCA total score is computed by summing up the score on each domain. The Chinese Beijing version (MoCA-ChiB) was developed based on a cohort of patients with mild cognitive impairment [34]. Sixteen items in the original English version were translated into Chinese. At the recommended cut-off score of 26, MoCA-ChiB yielded a sensitivity of 90.4% and a specificity of 31.3%. Optimal sensitivity (68.7%) and specificity (63.9%) were found at a cut-off of 22. The Cronbach's alpha of the MoCA-ChiB was 0.88, indicating good internal consistency. In this study, the MoCA-ChiB was utilized as an external criterion to establish the criterion validity evidence for the OCS-P.

#### 2.4.3. Goldenberg's Test

Goldenberg's test is a screening tool used to detect apraxia [35]. The test was designed based on the symptoms of ideomotor apraxia, and it requires the patient to attempt to perform three different gestures: imitation of hand postures, finger postures, and combined gestures. Each of these gestures should not be familiar to the patient. The patient was asked to imitate using the hand ipsilateral to the lesion. The maximum score for each gesture is 2 and the total score of the test is 12. The test was utilized to establish criterion validity evidence for the Praxis test in the OCS-P, which involves the imitation of hand-head and finger–hand postures.

### 2.5. Data Analysis

Item scores (mean, median, and 25th and 75th percentiles) and item-total (subscale score) correlations of the OCS-P were computed, providing evidence for the substantive validity. Construct validity was established by computing Spearman-rank correlations between scores of Goldenberg's test and the OCS-P “Praxis” subscale, as well as the selected subscales of MoCA-ChiB and OCS-P. For structural validity, confirmatory factor analysis (CFA) was conducted to examine the dimensionality of the OCS-P. The comparative fit index (CFI), standardized root mean square residual (SRMR), and root mean square error of approximation (RMSEA) were used to test the model fit. The values for acceptable fit were set at >0.90 for the CFI and <0.08 for the SRMR and RMSEA [36, 37]. For reliability, depending on the dimensionality revealed by the CFA, internal consistency using Cronbach's alpha coefficient was computed for each of the revealed dimensions. Potential differences in the two sets of the item scores were tested using Wilcoxon signed-rank test and Bonferroni correction was applied to adjust the *p* value to 0.005 due to multiple comparison. Intraclass correlation coefficient (ICC) was then used to estimate the interrater and intrarater reliability coefficient at the subscale and dimensional levels. Calculation of ICCs included estimation of their 95% confidence interval using a 2-way mixed-effect model and an agreement coefficient. Standard error of measurement (SEM) was calculated for both types of reliability coefficients at the subscale level following the formula: standard deviation of the subscale score multiplied by √(1 − *r*) where *r* is the ICC [38]. The 90% confidence interval minimal detectable change (MDC_90_) was estimated also at the subscale level. The formula used was MDC_90_ = 1.65 × √2 × SEM [39]. To test the known-group differences of OCS-P subscales, two-tailed *t*-tests were used with the significance level that was set at *p* ≤ 0.005 (Bonferroni adjustment for 10 subscales). Binary logistic regression was used to test the between-group discrimination of the OCS-P subscale scores between the patient and healthy older groups. Sensitivity and specificity and odds ratio (OR) of the significant subscales were used to determine the cut-off scores. Robustness of the between-group discrimination was tested by repeating the procedure to patient subgroups (hemorrhage versus ischemic and left- versus right-sided hemiplegia). All statistical analyses were carried out using SPSS version 20.0 (SPSS Inc., Chicago, IL, USA).

## 3. Results

One hundred poststroke patients were recruited from a post-acute rehabilitation hospital located in the southern part of mainland China (Table 1). Mean patient age was 59.3 years (SD = 8.8) with a mean educational level of 8.9 years (SD = 3.4). All patients were diagnosed with a first stroke (time from onset: 38.8 days (SD = 22.8)). Among the patients, 30 had hemorrhage stroke (14 left-sided and 16 right-sided hemiplegia) and 70 had ischemic stroke (42 left-sided and 28 right-sided hemiplegia) (Table 1). There were two healthy control groups: younger (*n* = 60; 55% female; mean age = 29.0 years (SD = 3.4)) and older (*n* = 60; 47% female; mean age = 58.7 years (SD = 6.5)). All the groups had comparable gender compositions and educational levels (Table 1). No significant differences in the demographic characteristics were revealed among the four patient subgroups, except that the left hemiplegic (hemorrhage) subgroup was significantly younger than the right hemiplegia (ischemic) subgroup (*p* = 0.023). The two right hemiplegia subgroups in general showed significantly lower mean MoCA total scores than the two left hemiplegia subgroups (*p* < 0.001).

### 3.1. Content Validity

The original English version of the “Sentence Reading” subscale was a 15-word sentence, which included four critical irregular words and four high neighborhood words. The Chinese version had 20 characters because of the single phoneme for each Chinese character which would make the length of the spoken Chinese sentence more comparable to that of the English version. The 20 characters incorporated regular/irregular words (or phrases), consistent/inconsistent phonetic-semantic compound characters (i.e., replacing highly neighborhood words that do not exist in Chinese), sentence structure, and the familiarity of the words (phrases) [40–42]. The research team maintained close communication with late Professor G. Humphreys (author of OCS) throughout the translation process to ensure the accuracy of the translation based on the aforementioned criteria. Back translation and reviews were conducted for all translated items, except for the “Sentence Reading” and “Delayed Recall & Recognition” subtests as the context and number of characters in the Chinese version are different from those in the English version. To establish the between-version comparability, we evaluated the structural validity and known-group discrimination of these subtests. Findings of the panel review revealed high content and linguistic equivalence for the test instructions in both English and Chinese (Putonghua) versions of the OCS. Evaluations of the content representativeness did not reveal specific issues between two versions. However, evaluations of content relevance revealed issues with four subscales, including “Picture Naming”, “Orientation”, “Sentence Reading”, and “Delayed Recall and Recognition”. Content irrelevance was related to cultural or linguistic differences between two versions. Panel members recommended modifications. Detailed modifications of the subscales can be found in the Supplementary Materials (available here) of this paper. 31 poststroke patients (9 females, mean age = 59.4) participated in the pilot field test. The score profiles and feedback from the patients did not reveal major issues on the level of fluency and understandability of the translated items. All the items reviewed by the expert panel were adopted in the final version of OCS-P for the main study.

### 3.2. Structural Validity

**Figure 1:**
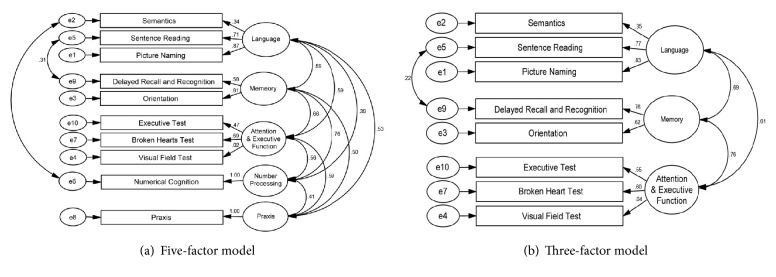
*Path diagram and estimated parameter loadings for the five- and three-factor models of the subscales of OCS-P*.* Note*. (a) The five domains proposed in the original OCS are attention (executive task, Broken Hearts test, and Visual Field Test), memory (orientation, delay recall, and recognition), language (semantics, Sentence Reading, and Picture Naming), number (Numerical Cognition), and Praxis. (b) The final three-factor model excluded the two single-subscale factors, which are “Numerical Cognition” and “Praxis”.

Based on the five-domain structure proposed in the original OCS [6], the initial CFA results indicated an unacceptable data-to-model fit (CFI = 0.89, SRMR = 0.07, and RMSEA = 0.11) (Figure 1(a)). To further improve the fitting, two paths which represent the correlations between the error terms of “Semantics” and “Numerical Cognition” and of “Sentence Reading” and “Delayed Recall and Recognition” were added in the CFA rerun. The modified model showed improvements in the fit to a nearly acceptable level (CFI = 0.92, SRMR = 0.06, and RMSEA = 0.09). To further tackle the RMSEA, the two single-subscale factors (i.e., “Numerical Cognition” and “Praxis”) were dropped from the model. This yielded an acceptable fit in the three-factor model (CFI = 0.96, SRMR = 0.05, and RMSEA = 0.06, Figure 1(b)). The three-factor model corresponded to the three domains stipulated in the OCS. The first dimension was the attention domain composed of “Executive Task”, “Broken Heart Test”, and “Visual Field Test” subscales. The second dimension was the memory domain composed of “Delayed Recall and Recognition” and “Orientation” subscales. The third dimension was the language domain composed of “Semantics”, “Sentence Reading”, and “Picture Naming” subscales.

### 3.3. Substantive Validity

Item scores and their mean, median, 25% tile, and 75% tile are summarized in Table 2. No missing item was revealed in the dataset. Subscales which showed possible ceiling effect for the patients were “Semantic” and “Visual Field Test” subscales, while no obvious flooring effect was observed. The subscale difficulty levels ranged from 0.59 to 0.97 (Table 3). The most difficult subscale was “Picture Naming” (mean = 0.59) whereas the easiest subscale was “Visual Field Test” (mean = 0.97). The discriminative index (or item-total correlation) was correlation between the subscale score and the total score of the dimension revealed by CFA. Discriminative indices were −0.72 to 0.32 for the attention dimension, 0.46 for the memory dimension, and 0.34 to 0.66 for the language domain (Table 3). No discriminative indices were yielded for the “Numerical Cognition” and “Praxis” subscales because they were both single-subscale dimensions.

### 3.4. Construct Validity

Correlation coefficients between the subscale scores of OCS-P and MoCA-ChiB were largely moderate to high (*r* = 0.45–0.79, *p* < 0.001) (Table 4). The correlation between the scores of Goldenberg's test and the Praxis subscale of OCS-P was high (*r* = 0.72, *p* < 0.001).

### 3.5. Reliability

Internal consistency indices of the attention, memory, and language dimensions were 0.30, 0.52, and 0.44, respectively (Table 3). No significant differences were revealed in the OCS-P subscale scores between the two raters for interrater reliability (1.000 ≥ *p* ≥ 0.020) and within the same rater between two assessments in a one-week interval for intrarater reliability (1.000 ≥ *p* ≥ 0.014). Except for the “Praxis” subscale, excellent interrater reliability coefficients were revealed in all other OCS-P subscales. Moderate to excellent intrarater reliability coefficients were revealed in all subscales (Table 5). The SEMs estimated for the subscales based on the interrater and intrarater reliability coefficients varied according to the standard deviations of the subscale scores and the values of the coefficients. Among them, the “Broken Heart Test”, “Semantics”, and “Praixa” subscales had larger SEMs. Similarly, these subscales had larger MCD_90_ than the other subscales.

### 3.6. Known-Group Validity

Healthy young participants obtained relatively higher scores for most OCS-P subscales when compared with healthy older participants (Table 2). Only the “Orientation” and “Sentence Reading” subscales showed significant differences (after Bonferroni adjustment). Poststroke patients showed significantly lower scores in almost all subscales when compared with the healthy older participants. Those subscales with the largest differences were “Picture Naming”, “Delayed Recall”, and “Recognition-Verbal Recall”.

As shown in Table 6, logistic regression revealed four significant subscales predicting the two group memberships (patients versus healthy older). The Nagelkerke's *R*^2^ value (0.793) indicated strong association between predictors and participants' membership [43]:

(1) Y=48.65−1.58 Picture  Naming−3.45 Numerical  Cognition−0.72 Praxis−1.51 Delayed  Recall  and  Recognition

[Note: values of *Y*: 1 = poststroke patients and 0 = healthy controls)].

**Figure 2:**
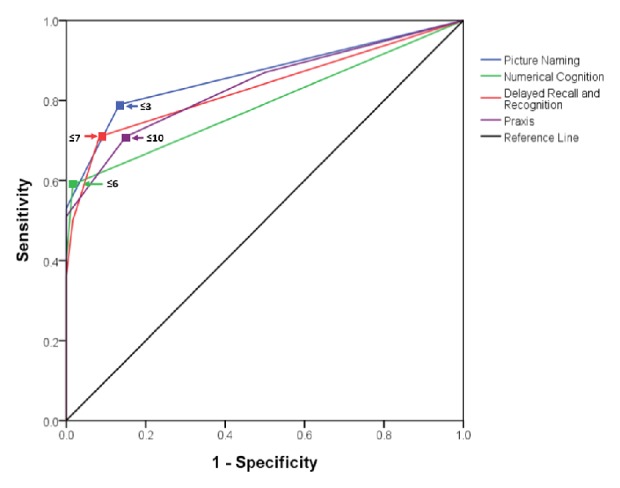
*The receiver-operating characteristic (ROC) curves of “Picture Naming”, “Numerical Cognition”, “Praxis”, and “Delayed Recall and Recognition” for discriminating poststroke patients from the healthy older counterparts*. Arrows indicate the corresponding optimal cut-off score of each subscale.

 Receiver-operating characteristics (ROC) analysis indicated that all subscales produced satisfactory areas under the curve (AUCs), ranging from 0.790 (Numerical Cognition) to 0.864 (Picture Naming) (Figure 2). “Picture Naming” was found to produce the most optimal sensitivity (79%) and specificity (86.7%) for the cut-off of 3 out of 4 (OR = 24.5 with 95% CI = 10.1–59.3). In contrast, the “Numerical Cognition” yielded a lower sensitivity (59.0%) and a higher specificity (98.3%) for a cut-off score of 6 out of 7 (OR = 84.9; 95% CI, 11.3–637.6).

The patient participants were further divided into four subgroups according to the side and type of brain lesions. The right hemispheric subgroup had significantly lower scores (*p* < 0.005) in all subscales (Table 7). ROC analysis indicated that the subscales continued to produce satisfactory AUCs (Table 8). Consistent with earlier results, the highest AUCs were from “Picture Naming” (0.797 to 0.975), while the lowest AUCs were from “Numerical Cognition” (0.709 to 0.934). “Picture Naming” yielded the most optimal sensitivity and specificity for the cut-off of 3 (out of 4) for the left versus right ischemic subgroups. In contrast, “Praxis” was the most optimal for the cut-off of 10 (out of 12) for the left hemorrhage versus left ischemic subgroups.

## 4. Discussion

Our findings suggest that the eight subscales of OCS-P showed a three-dimension test structure, which resembles the attention, memory, and language domains proposed in the original OCS [6]. Confirmatory factor analysis results did not support acceptable data-to-model fit when the other two single-subscale domains were taken into consideration. Cronbach's alpha values estimated for the internal consistency at the dimension level, however, were relatively low. As this is the first paper reporting the structural validity of OCS, more studies should test further hypothesis on the test construct of the instrument. The strong relationships revealed between the OCS-P subscales and their corresponding MoCA-ChiB subscales support our hypothesis that the translated version possessed good construct validity. Besides, the results indicate that OCS-P had excellent to good interrater reliability and good to fair intrarater reliability. The known-group analyses demonstrate that OCS-P was able to differentiate poststroke patients from healthy controls. Extending this membership prediction to brain lesion subgroups, the OCS-P demonstrates a similar level of prediction accuracy. These results provide support for the OCS-P as a useful screening test for assessing cognitive functioning of poststroke patients. The findings on the structural validity, data-to-model misfit of the Numerical Cognition and Praxis subscales, prompt further review of the five-factor domains stipulated in the original OCS. That said, its primary development aim was to briefly screen for cognitive impairments which may affect patients' rehabilitation care pathways, and the impact of apraxia and inability to write, as assessed by the OCS, are important aspects to highlight.

The OCS-P was translated in accordance with the requirements of the Chinese language. Moreover, the content was modified to accommodate the specificity of Chinese culture, while maintaining its equivalence to the content of the original version. This is supported by the confirmatory factor analysis results that the language domain proposed in the original OCS comprising “Semantics”, “Sentence Reading”, and “Picture Naming” subscales was replicated. Poststroke patients scored significantly lower than the healthy older participants on these subscales. It is noteworthy that, in the course of analysis, a reasonable model-data fit can only be achieved after correlating the error term between “Sentence Reading” and “Delayed Recall and Recognition” and “Semantics” and “Numerical Cognition”. These significant correlated error terms may suggest lack of independence between the two pairs of subscales. For instance, patients would need to learn the sentence well when performing in “Sentence Reading” subscale before they could recall the sentence when performing in “Delayed Recall and Recognition” subscale. The interdependency of learning the sentence first and then recalling it later perhaps can explain the significant correlations between the two subscales. On the same token, the computational processes tapped in “Numerical Cognition” subscale could involve understanding of the meanings of numbers and mathematic operations which overlaps with the content of “Semantics” subscale. This calls for future studies to investigate the ways to reduce the interdependency of the language domain subscales with other nonlanguage subscales and hence improve their psychometric properties.

Moderate to high correlations were yielded in most of the subscales between OCS-P and MoCA-ChiB, suggesting good construct validity for the OCS-P. Evidence on the construct validity of the OCS-P is comparable to those for the original OCS [6, 44].

The internal consistency estimated for each of the attention, memory, and language dimensions (or domain) was of low values, ranging from 0.30 to 0.52. These low values suggest that the correlations among the subscale scores within a dimension tended to be low. Our findings are inconsistent with those reported in the original and Cantonese (or HK-OCS) OCS. No internal consistency was reported in the original OCS [6]. The HK-OCS reported a single internal consistency index, which was 0.725 at the total test level [24]. The interrater reliability coefficients yielded for OCS-P were very high, which are comparable to those reported for HK-OCS. The intrarater reliability coefficients obtained were moderate to excellent, which are higher than those for the original OCS (test-retest reliability). The lower intrarater reliability coefficients yielded could have been due to the unavoidable changes in the cognitive functions due to intensive rehabilitation interventions which the patients received while staying in the hospital. These changes would have contributed to the inconsistencies in the two sets of scores entered into the intraclass correlation computation. The errors of measurement (SEMs) and minimal detectable changes (MDCs) estimated based on the interrater reliability coefficients for OCS-P therefore were zero or small in values. The only values obtained were those for the “Praxis” subscale of which the SEM was around 0.5 and the MDC was around 1.3 (out of maximum 12). In contrast, those estimated based on the intrarater reliability coefficients were larger. The smallest values were found in the “Visual Field Test” subscale which was zero for both the SEM and MDC, respectively. The largest values were in the “Semantics” subscale of around 0.3 and 0.8 (out of maximum 3) for the SEM and MDC, respectively. The choice of SEM and MDC should depend on the purpose of using OCS-P. The interrater reliability coefficients and their SEMs and MDCs would be more relevant for reference when the test is used for the screening of specific poststroke cognitive deficits. The intrarater reliability coefficients and their SEMs and MDCs would be more relevant for use when the test is employed for measuring changes in cognitive functions during poststroke rehabilitation.

The poststroke patients scored relatively higher on the “Visual Field”, “Semantics”, “Orientation”, and “Broken Hearts” subscales and lower on “Picture Naming”, which are comparable to those reported in the original OCS validation study [6]. This study adopted a rather stringent criterion (*p* ≤ 0.005) for testing the significance of between-group differences in OCS-P subscale scores. Poststroke patients scored significantly lower than the older healthy participants in all except the “Visual Field Test”. These findings indicate that the design of the content and difficultly level of test items of the OCS-P are appropriate for poststroke patients. Among all subscales, four were revealed to be effectively differentiating the poststroke patients from the older healthy participants. They were “Picture Naming”, “Numerical Cognition”, “Delayed Recall and Recognition”, and “Praxis”. These subscales were developed to measure the level of expressive language, number processing, verbal memory, and skilled action, respectively [6, 45]. Besides the OCS, naming task is commonly found in other clinical instruments such as MoCA, MMSE, and Cognistat [45–48]. The task involves primarily retrieval of semantic knowledge about the object and access to the phonological representation for articulation [49, 50]. The significant finding of “Picture Naming” supports the notion that the OCS-P has strong language evaluation capacity.

In contrast to “Naming Picture”, “Numerical Cognition” was identified as a strong predictor of poststroke patient membership with high specificity but rather low sensitivity (59.0%). This finding is comparable to that reported in the original OCS validation study [6]. The “Praxis” subscale was found to yield the most optimal sensitivity and specificity for identifying patients with left hemiplegia in this study. One drawback of the “Praxis” subscale is the relatively low interrater reliability revealed in this study and the HK-OCS validation study [24]. Future study should improve the objectivity of its scoring criteria so as to improve the interrater reliability. The “Delayed Recall and Recognition” subscale was found to possess comparable discriminative power with the “Praxis” subscale.

There are several limitations to our study. First, poststroke patients recruited in this study differed in education level and onset time from those who were involved in the original OCS study. These discrepancies may have resulted in score differences obtained for poststroke patients between these studies. Second, the number of patients involved in the inter- and intrarater reliability was rather small. The relatively small sample size could have weakened the power of the statistical analyses. Readers should be cautious when interpreting the results. Besides, the number of patients was small for conducting the subgroup analyses, which might not adequately power the subgroup differentiation. Patients in this study did not include individuals who presented with neglect problems. Thus, the psychometric properties reported for OCS-P cannot be generalized to poststroke patients who present with a visual neglect problem. Future studies should compare the OCS-P scores between poststroke patients with and without neglect problems.

## 5. Conclusions

It is important to accurately detect cognitive impairments in stroke rehabilitation. OCS-P was appropriate for use as a cognitive screening tool for poststroke nonneglect patients who spoken Putonghua. The results revealed the OCS-P has satisfactory content validity, substantive validity, construct validity, inter- and intrarater reliability, and known-group discrimination. Future studies could review the five-dimension domains stipulated in the original OCS to further improve the structural validity and hence internal consistency of the instrument. Besides, the clinical utility of the OCS-P for predicting functional recovery and discharge plans of poststroke patients can be explored.

## Figures and Tables

**(a):** 

Total Group	Post-stroke Patients (*N* = 100)	Younger Healthy Participants (*N* = 60)	Older Healthy Participants (*N* = 60)	*p*-value^*∗*^
Age (Mean ± SD)	59.2 ± 8.8	29.0 ± 3.4	58.7 ± 6.6	<0.001
Gender (male) (%)	66	52	53	0.127
Education (years ± SD)	8.9 ± 3.4	10.0 ± 2.7	9.5 ± 2.8	0.085
MoCA (Mean ± SD)	16.1 ± 5.8	-	-	NA
Time since event (Mean ± SD)	38.8 days ± 22.8	-	-	NA

*Note*. MoCA = Montreal Cognitive Assessment; ^*∗*^between-group comparisons were conducted using one-way ANOVA or Chi-square test (only for gender). “NA” means not applicable.

**(b):** 

Patient Sub-groups	Hemorrhage		Ischemic		*p*-value
	Left Hemi.	Right Hemi.	Left Hemi.	Right Hemi.	
	(*n* = 14)	(*n* = 16)	(*n* = 42)	(*n* = 28)	
Age (Mean ± SD)	54.6 ± 9.1	56.6 ± 9.2	60.1 ± 9.0	62.5 ± 7.2	0.023
Gender (female/male)	6/8	3/13	18/24	7/21	0.201
Education (years ± SD)	9.0 ± 3.9	9.0 ± 3.0	8.9 ± 3.6	8.7 ± 3.2	0.990
MoCA (Mean ± SD)	17.0 ± 4.8	12.8 ± 5.1	19.0 ± 5.1	13.0 ± 5.4	<0.001

*Note*. Hemi. = hemiplegia. MoCA = Montreal Cognitive Assessment; ^*∗*^between-group comparisons were conducted using one-way ANOVA or Chi-square test (only for gender).

**Table 2:** Summary of item scores and their means, median, 25% tile, and 75% tile. Results of comparisons of the OCS-P subscale scores among patient and healthy groups.

Subscale scores on OCS-P Mean + SD (25% tile, median, 75% tile)	Post-stroke patients (PS) (*N* = 100)	Healthy Younger Participants (Y) (*N* = 60)	Healthy Older Participants (O) (*N* = 60)	Differences	
				(*p* values^a^)	
				PS vs O	Y vs O
Picture Naming	2.4 ± 1.2	4.0 ± 0.2	3.9 ± 0.3	<0.001	0.048
	(2.0,2.0,2.0)	(4.0,4.0,4.0)	(4.0,4.0,4.0)		
Semantics	2.8 ± 0.5	3.0 ± 0.0	3.0 ± 0.2	0.007	0.156
	(3.0,3.0,3.0)	(3.0,3.0,3.0)	(3.0,3.0,3.0)		
Orientation	3.5 ± 0.8	3.6 ± 0.8	4.0 ± 0.0	<0.001	<0.001
	(3.0,4.0,4.0)	(4.0,4.0,4.0)	(4.0,4.0,4.0)		
Visual Field Test	3.9 ± 0.5	4.0 ± 0.0	4.0 ± 0.0	0.037	NA
	(4.0,4.0,4.0)	(4.0,4.0,4.0)	(4.0,4.0,4.0)		
Sentence Reading	16.2 ± 4.2	19.5 ± 0.6	19.0 ± 0.9	<0.001	0.001
	(15,18,19)	(19,20,20)	(18,19,20)		
Numerical Cognition					
Writing	2.3 ± 1.0	3.0 ± 0.2	3.0 ± 0.0	<0.001	0.156
	(2.0,3.0,3.0)	(3.0,3.0,3.0)	(3.0,3.0,3.0)		
Calculations	3.2 ± 1.1	4.0 ± 0.1	4.0 ± 0.1	<0.001	NA
	(2.0,4.0,4.0)	(4.0,4.0,4.0)	(4.0,4.0,4.0)		
Total	5.4 ± 1.8	7.0 ± 0.2	7.0 ± 0.1	<0.001	0.311
	(4.0,6.0,7.0)	(7.0,7.0,7.0)	(7.0,7.0,7.0)		
Broken Heart Test					
TC	42.6 ± 6.4	48.7 ± 1.8	48.2 ± 2.2	<0.001	0.285
	(38,44,48)	(48,49,50)	(47,49,50)		
SA	0.1 ± 1.9	−0.2 ± 0.8	0.0 ± 0.9	0.560	0.539
	(−1.0,0, 1.0)	(−1.0,0, 0)	(−1.0,0, 0)		
OA	1.0 ± 4.4	0.0 ± 0.3	−0.0 ± 0.2	0.001	0.104
	(0,0, 1.0)	(0,0, 0)	(0,0, 0)		
Praxis	9.1 ± 2.1	11.7 ± 0.5	11.4 ± 0.7	<0.001	0.020
	(8.0,8.0,11)	(11,12,12)	(11,11.5,12)		
Delayed Recall & Recognition					
VR	0.7 ± 1.0	3.2 ± 0.7	2.2 ± 0.8	<0.001	<0.001
	(0,0, 1.0)	(3.0,3.0,4.0)	(2.0,2.0,3.0)		
VRR	2.9 ± 1.1	4.0 ± 0.1	4.0 ± 0.1	<0.001	NA
	(2.0,3.0,4.0)	(4.0,4.0,4.0)	(4.0,4.0,4.0)		
ER	3.3 ± 0.8	4.0 ± 0.0	3.9 ± 0.3	<0.001	0.023
	(3.0,3.0,4.0)	(4.0,4.0,4.0)	(4.0,4.0,4.0)		
Total	6.2 ± 1.7	8.0 ± 0.1	7.9 ± 0.4	<0.001	0.094
	(5.0,6.5,8.0)	(8.0,8.0,8.0)	(8.0,8.0,8.0)		
Executive Task					
Mixed	8.5 ± 3.4	12.5 ± 1.6	12.4 ± 1.8	<0.001	0.232
	(8.0,6.0,12)	(13,13,13)	(13,13,13)		
Total^b^	2.7 ± 3.2	−0.5 ± 1.6	−0.5 ± 1.8	<0.001	0.697
	(0,3.0,5.0)	(−1.0, −1.0, −1.0)	(−1.0, −1.0, −1.0)		

*Note*. TC, total correct; SA, space asymmetry; OA, object asymmetry; VR, verbal recall; VRR, verbal recall and recognition; and ER, episodic recognition. “NA” means not applicable; ^a^*p* values are results of independent *t*-tests. ^b^Total = Circle + Triangle – Mixed.

**Table 3:** Difficulty levels, discriminative indices (item-total correlations), and internal consistency (Cronbach's alpha) of the OCS-P subscales according to attention, memory, and language dimensions.

OCS-P subscales (*N* = 100)	Difficulty level	Discriminative index	Internal consistency (Cronbach's alpha)
Visual Field Test	0.97	−0.72	-
Broken Hearts Test	0.85	0.32	-
Executive Task	0.65	0.31	-
[Attention Dimension]			0.30

Orientation	0.87	0.46	-
Delayed Recall & Recognition	0.76	0.46	-
[Memory Dimension]			0.52

Semantics	0.93	0.34	-
Sentence Reading	0.81	0.66	-
Picture Naming	0.59	0.64	-
[Language Dimension]			0.44

Numerical Cognition	0.77	NA	-
Praxis	0.75	NA	-

*Note*. Difficulty level means patients' scores on the subscale divided by the maximum subscale score. The three test dimensions are attention, memory, and language. Discriminative index (item-total correlation) was correlation between the subscale score and the total score of the dimension. “NA” means not applicable because the subscale score is the same as the dimension score.

**Table 4:** Pearson's correlation coefficients between OCS-P and MoCA-ChiB subscales.

OCS-P subscales	MoCA-ChiB subscales	*r*	*p* values
Picture Naming	Naming	0.73	<0.001
Orientation	Orientation	0.68	<0.001
Calculations	Serial 7 Subtraction	0.79	<0.001
Sentence Reading	Language	0.55	<0.001
Verbal memory - Free recall	Delayed Recall	0.45	<0.001
Executive task-Mixed	Trails	0.67	<0.001

**Table 5:** Interrater and intrarater reliability coefficients of the OCS-P.

OCS-P subscales	Inter-rater reliability^*∗*^	Intra-rater reliability^*∗*^	SEM/MDC_90_	SEM/MDC_90_
	(*n* = 15)	(*n* = 15)	(inter-rater)	(intra-rater)
Visual Field Test	1.00	1.00	0.00/0.00	0.00/0.00
Broken Hearts Test	1.00	0.79	0.00/0.00	2.64/6.17
		(0.50, 0.93)		
Executive Task	1.00	0.82	0.00/0.00	1.31/3.05
		(0.52, 0.94)		
[Attention Dimension]	1.00	0.85	0.00/0.00	1.98/4.62
		(0.55, 0.95)		

Orientation	1.00	0.83	0.00/0.00	0.41/0.96
		(0.59, 0.94)		
Delayed Recall & Recognition	1.00	0.80	0.00/0.00	0.64/1.49
		(0.51, 0.93)		
[Memory Dimension]	1.00	0.93	0.00/0.00	0.59/1.37
		(0.78, 0.98)		

Semantics	1.00	0.58	0.00/0.00	0.34/0.80
		(0.13, 0.84)		
Sentence Reading	1.00	0.98	0.00/0.00	0.75/1.74
		(0.94, 0.99)		
Picture Naming	1.00	0.97	0.00/0.00	0.24/0.55
		(0.90, 0.99)		
[Language Dimension]	1.00	0.99	0.00/0.00	0.67/1.56
		(0.96, 1.00)		

Numerical Cognition	1.00	0.92	0.00/0.00	0.66/1.53
		(0.63, 0.98)		
Praxis	0.90	0.76	0.53/1.24	0.88/2.06
	(0.61, 0.97)	(0.42, 0.91)		

*Note*. The three test dimensions are attention, memory, and language. ^*∗*^Intraclass correlation coefficients (ICCs) and values in brackets are lower and upper bounds of the 95% confidence interval. SEM is standard error of measurement. MDC_90_ is minimal detectable change with 90% confidence interval.

**Table 6:** Results of binary logistic regression and the ROC analysis of four OCS-P subscales (with cutoff scores) for discriminating poststroke patients from the healthy older participants.

Variables/OCS-P Subscales (optimal cutoff)	*β*	SE	Wald	*p* values	Sensitivity	Specificity	AUC	OR (95% CI)
Constant	48.65	10.87	20.04	<0.001	-	-	-	-
Picture Naming (⩽3)	−1.58	0.54	8.57	0.003	0.79	0.87	0.86	24.45 (10.08, 59.33)
Numerical Cognition (⩽6)	−3.45	1.23	7.92	0.005	0.59	0.98	0.79	84.90 (11.30, 637.67)
Praxis (⩽10)	−0.72	0.29	6.26	0.012	0.71	0.85	0.84	13.87 (6.05, 31.81)
Delayed Recall and Recognition (⩽7)	−1.51	0.56	7.36	0.007	0.71	0.92	0.83	26.93 (9.79, 74.11)

*Note*. Cut-off scores are based on optimal sensitivity and specificity for each subscale. SE, standard error; AUC, area under the curve; OR, odds ratio; and CI, confidence interval.

**Table 7:** Comparisons of the subscales of Picture Naming, Numerical Cognition, Praxis, and Delayed Recall and Recognition subscales among four patient subgroups.

Subscale scores on OCS-P (Mean + SD)	Hemorrhage		Ischemic		*p*-value^*∗*^
	Left Hemi.	Right Hemi.	Left Hemi.	Right Hemi.	
	(*n* = 14)	(*n* = 16)	(*n* = 42)	(*n* = 28)	
Picture Naming	2.43 ± 1.09	2.00 ± 1.03	2.69 ± 1.16	1.86 ± 1.24	0.022
Numerical Cognition	5.71 ± 1.68	4.19 ± 2.04	5.55 ± 1.85	5.43 ± 1.67	0.060
Praxis	8.79 ± 1.89	8.38 ± 2.63	9.36 ± 1.90	8.86 ± 2.35	0.439
Delayed Recall & Recognition (Total)	5.79 ± 2.01	5.19 ± 1.97	6.83 ± 1.31	5.71 ± 1.72	0.003

*Note*. Hemi. = hemiplegia. ^*∗*^Between-group comparisons were conducted using one-way ANOVA.

**Table 8:** Comparisons of areas under the curve (AUC) across the four patient subgroups of OCS-P Picture Naming, Numerical Cognition, Praxis, and Delayed Recall and Recognition Subscales.

Subscales	Hemorrhage		Ischemic	
	Left Hemi.	Right Hemi.	Left Hemi.	Right Hemi.
Picture Naming (⩽3)	0.864	0.975	0.797	0.940
Numerical Cognition (⩽6)	0.709	0.934	0769	0.817
Praxis (⩽10)	0.877	0.852	0.823	0.850
Delayed Recall and Recognition (⩽7)	0.872	0.922	0.754	0.878

*Note*. Hemi. = hemiplegia.

## Supplementary Materials

Supplementary Materials Summary of the modifications made to the content of the OCS as a result from the evaluation of the content validity and panel review for the OCS-P. It includes two items in “Picture Naming”, two items in “Orientation”, the sentence in “Sentence Reading”, and the targets and distractors in the verbal memory and episodic memory sections in “Delayed Recall and Recognition”.
