# Supplementary material for: Psychometric Properties of the Chinese (Putonghua) Version of the Oxford Cognitive Screen (OCS-P) in Subacute Poststroke Patients without Neglect
Source: Biomed Res Int. 2018 May 21;2018:6827854. doi: 10.1155/2018/6827854 (PMC5987308; doi:10.1155/2018/6827854)
Supplement: Supplementary Materials — Summary of the modifications made to the content of the OCS as a result from the evaluation of the content validity and panel review for the OCS-P. It includes two items in “Picture Naming”, two items in “Orientation”, the sentence in “Sentence Reading”, and the targets and distractors in the verbal memory and episodic memory sections in “Delayed Recall and Recognition”. [file 6827854.f1.pdf]

Supplementary Materials

Modifications of content in four OCS subscales:

(1) ‘Picture Naming’. Two items were modified. In item 1, the animal, which was a hippopotamus, was changed to a “长颈鹿” (giraffe, *chang jing lu*) (See below). This modification was made because the hippopotamus was not commonly found in China; *chang jing lu* would share a similar level of familiarity and linguistic difficulty with the hippopotamus. In item 4, the shape and color of the pear was slightly adjusted to better reflect the type of pear that is commonly found in China.

Sample of Item 1 “Giraffe” in the Picture Naming subscale.

Picture Naming: Item 1 “长颈鹿”<br><br>(giraffe, *chang jing lu*)	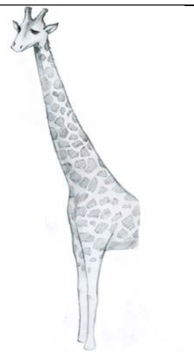

(2) ‘Orientation’. In item 1, changes were made to the names of four cities, “*Which city or town are we in*?” *(我们现在在哪个城市? Wo men xian zai na ge cheng shi?)*. The city names of Oxford, Cambridge, Reading, and Warwick were changed to 福州 (*Fuzhou*), 上海 (*Shanghai*), 北京 (*Beijing*), and 佛山 (*Foshan*). *Shanghai* and *Beijing* in China are two major cities deemed to be comparable to Cambridge and Warwick in the United Kingdom. *Foshan* is a smaller city that is more comparable to Reading. Oxford and *Fuzhou* are the cities where the English and Chinese versions of OCS were developed, respectively. In Item 4, the years 2013, 2012, and 2011 were replaced with the years 2015, 2014, and 2013 to better reflect the current year.

(3) ‘Sentence Reading’. The English version included a fifteen-word sentence, in which four words were critical irregular words and four words were high neighborhood words. The critical irregular words were “islands”, “quay”, “colonel”, and “yacht”. The high neighborhood words were “have”, “any”, “islands”, and “thoughts”. The Chinese version included the 20-character sentence that was organized into three rows: “赌局的气氛颇浓 / 间谍小菲只嫌 / 乌鸦聚拢麻烦多” (The gambling atmosphere was quite intense/ which the spy *Xiaofei* dislikes/ because complicated crowds would gather and could stir up much trouble; *du ju de qi fen po nong* / *jian die iao fei zhi xian* / *wu ya ju long ma fan duo*). The four irregular character phrases were: ”赌局” (gambling, *du ju*), ”间谍” (spy, *jian die*), “乌鸦” (complicated crowds, *wu ya*), and ”麻烦” (trouble, *ma fan*).

(4) ‘Delayed Recall and Recognition’. This subtest had two parts, one was used to test for verbal memory with reference to the ‘Sentence Reading’ section and the other was for episodic memory with reference to the ‘Picture Naming’ section. For verbal memory, four irregular character phrases were the targets, while three distractors were constructed for each irregular phrase according to the criteria established in the original test (i.e., one semantically similar character phrase and two unrelated phrases). For episodic memory, the revised items in the ‘Picture Naming’ section replaced those found in the original version (see #1 above).
